# Modification of the association between maternal smoke exposure and congenital heart defects by polymorphisms in glutathione S-transferase genes

**DOI:** 10.1038/srep14915

**Published:** 2015-10-12

**Authors:** Xiaohong Li, Zhen Liu, Ying Deng, Shengli Li, Dezhi Mu, Xiaoxian Tian, Yuan Lin, Jiaxiang Yang, Jun Li, Nana Li, Yanping Wang, Xinlin Chen, Kui Deng, Jun Zhu

**Affiliations:** 1 National Center for Birth Defect Monitoring of China, West China Second University Hospital, Sichuan University, Chengdu, China; 2 Department of Epidemiology and Health Statistics, West China School of Public Health, Sichuan University, Chengdu, Sichuan, China; 3 Laboratory of Molecular Epidemiology for Birth Defects, West China Institute of Women and Children’s Health, West China Second University Hospital, Sichuan University, Chengdu, China; 4 Department of Ultrasound, Shenzhen Maternity & Child Healthcare Hospital, Southern Medical University, Shenzhen, Guangdong, China; 5 Department of Pediatrics, West China Second University Hospital, Sichuan University, Chengdu, 610041, China; 6 Department of Ultrasound, Maternal and Child Healthcare Hospital of Guangxi Zhuang Autonomous Region, Nanning, Guangxi, China; 7 Department of Obstetrics & Gynecology, Fujian Provincial Maternal and Child Healthcare Hospital, Fuzhou, Fujian, China; 8 Department of Ultrasound, Sichuan Provincial Maternal and Child Healthcare Hospital, Chengdu, Sichuan, China; 9 Department of Ultrasound, Xijing Hospital, Fourth Military Medical University, Xi’an, Shanxi, China; 10 National Office for Maternal and Child Health Surveillance, West China Second University Hospital, Sichuan University, Chengdu, China; 11 Hubei Provincial Maternal and Child Healthcare Hospital, Wuhan, China; 12 Key Laboratory of Birth Defects and Related Diseases of Women and Children (Sichuan University), Ministry of Education, Chengdu, China

## Abstract

Congenital heart defects (CHDs) arise through various combinations of genetic and environmental factors. Our study explores how polymorphisms in the glutathione S-transferase (GST) genes affect the association between cigarette smoke exposure and CHDs. We analysed 299 mothers of children with CHDs and 284 mothers of children without any abnormalities who were recruited from six hospitals. The hair nicotine concentration (HNC) was used to quantify maternal smoke exposure, and the maternal GSTT1, and GSTM1 and GSTP1 genes were sequenced. We found a trend of higher adjusted odds ratios with higher maternal HNC levels, suggesting a dose-response relationship between maternal smoke exposure and CHDs. The lowest HNC range associated with an increased risk of CHDs was 0.213–0.319 ng/mg among the mothers with functional deletions of GSTM1 or GSTT1and 0.319–0.573 ng/mg among the mothers with normal copies of GSTM1 and GSTT1. In addition, the adjusted odds ratio for an HNC of >0.573 ng/mg was 38.53 among the mothers with the GSTP1 AG or GG genotype, which was 7.76 (χ^2^ = 6.702, *p* = 0.010) times greater than the AOR in the mothers with GSTP1 AA genotype. Our study suggests that polymorphisms of maternal GST genes may modify the association of maternal smoke exposure with CHDs.

The aetiology of congenital heart defects (CHDs), which are among the most common birth defects with an estimated prevalence of 6–8% of live births, is largely an enigma^1^. Although there has been substantial progress in recent years in understanding the genetic and chromosomal risk factors for CHDs^2^, relatively few non-inherited, modifiable risk factors for CHDs are known. Increasing evidence from epidemiological studies over the last two decades indicates that maternal smoking during pregnancy is an important risk factor for CHDs^3^,^4^. The global estimated prevalence of daily smoking was 31.1% for men and 6.2% for women in 2012^5^. Although most adult females in China do not smoke, more than half of the adult males are smokers, which results in approximately 300 million adult women exposed to the secondhand smoke^6^. Therefore, the study of the relationship between maternal smoke exposure and CHDs has great public health significance.

It is important to note that maternal smoke exposure identified in most previous studies was based on self-reporting by the women^3^,^7^. Self-reporting is particularly unreliable in ascertaining true smoke exposure because of the uncertainties in memory recall, the exposure to environmental tobacco smoke (ETS), and the social stigma of smoking during pregnancy^8^. Thus, it is important to identify a biological marker that objectively indicates maternal exposure to smoke. In recent years, hair nicotine analysis has emerged as a novel, non-invasive method to assess long-term exposure to tobacco smoke because nicotine can accumulate and remain stable in newly formed hair^9^,^10^,^11^.

Accumulating evidence suggests that the gene products responsible for activating and detoxifying xenobiotics can influence the effects of smoking on cancer, coronary artery disease, and congenital anomalies (e.g., oral clefts)^12^,^13^,^14^. The glutathione S-transferases (GST), which are part of an important family of dimeric phase II metabolic enzymes involved in the detoxification of xenobiotics, might modulate the adverse effects of exogenous toxins^15^,^16^. At least four main classes of soluble GSTs have been identified in humans: alpha (A), mu (M), pi (P), and theta (T), which are encoded by2 genes (GSTA1,A2, located at 6p12), 5 genes (GSTM1-M5, located at 1p13.3), 1 gene (GSTP1, located at 11q13) and 2 genes (GSTT1,T2, located at 22q11.2), respectively. Functional polymorphisms have been reported in the GSTT1, GSTM1, and GSTP1 genes. These include deletions in GSTT1 and GSTM1, which can abrogate enzyme activity, as well as an I105 V polymorphism in GSTP1, which is thought to result in diminished enzyme activity. It has been proposed that mothers with such GSTT1, GSTM1 or GSTP1 polymorphisms are less capable of effectively detoxifying environmental xenobiotics (such as tobacco smoke by-products) that cross the placenta; as a result, these mothers and their foetuses are exposed to elevated toxin levels^16^. Therefore, we hypothesized that GST gene polymorphisms may increase the susceptibility to cigarette smoke-related human diseases, including CHDs. One study indicated that polymorphisms in GSTM1 and GSTT1can potentially modify the risk of toxicant exposure-associated CHDs^17^, but there is little direct evidence regarding whether maternal GST gene polymorphisms can modify the risk of smoke exposure-associated CHDs. More studies are required to confirm this hypothesis.

In 2009, we initiated a study program, “Gene-Environment Interaction on CHDs” (GEIOC), to explore how environmental and genetic factors interact to influence CHDs in human offspring. The current study is an important part of the GEIOC. We explored the association between maternal smoke exposure and the occurrence of foetal CHDs and evaluated whether this association is modified by polymorphisms in GSTT1, GSTM1, or GSTP1.

## Results

**Figure 1:**
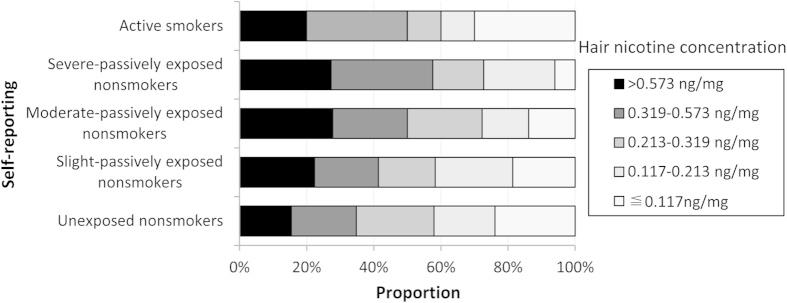
The distribution of HNC levels among the self-reporting groups.

The characteristics of the case and control groups are listed in Table 1. Maternal education level, alcohol drinking frequency, and pre-pregnancy body mass index (ppBMI) were significantly different between the case and control groups. The maternal hair nicotine concentration (HNC) showed a left-skewed distribution in the case and control groups (Table 2). In the case group, the geometric mean (GM) of the HNC was 0.369 ng/mg, which was higher than that of the control group (0.171 ng/mg) (t = −9.65, *p* < .0001). A large variation in HNC was observed within each self-reporting category (Fig. 1). The mothers with an HNC of >0.319 ng/mg accounted for 34.75%, 41.32%, 50.00%, 57.58%, and 50.00% of the unexposed nonsmokers, slight passively exposed nonsmokers, moderate passively exposed nonsmokers, severe passively exposed nonsmokers, and active smokers, respectively. No significant differences in the frequencies of GST gene polymorphisms were observed between these two groups.

Compared with an HNC of ≤0.117 ng/mg, the adjusted odds ratios (AOR) for HNCs of >0.573 ng/mg, 0.319–0.573 ng/mg, and 0.213–0.319 ng/mg were 6.83 (95% CI: 3.64, 12.80), 4.37 (95% CI: 2.42, 7.88), and 1.94 (95% CI: 1.09, 3.46), respectively. There also seemed to be a trend toward higher AOR with higher HNCs for each subcategory of CHDs (Table 3). Among the mothers with deletions in GSTM1 and/or GSTT1, the lowest HNC range associated with an increased risk of any CHDs was 0.213–0.319 ng/mg; in contrast, that range was 0.319–0.573 ng/mg among the mothers with normal copies of GSTM1 and GSTT1 (Table 4). Some AORs were higher among mothers without the favourable genotypes, but that was not a consistent finding across the GST genes and HNC levels. The AORs for the HNC levels of >0.213 ng/mg among the mothers with deletions in GSTM1 were higher than those among the mothers with normal copies of GSTM1. In addition, the AOR for an HNC of >0.573 ng/mg was 38.53 (95% CI: 8.17, 181.84) among the mothers with the GSTP1 AG or GG genotype. This AOR was 7.76 (χ^2^ = 6.702, *p* = 0.010) times greater than the AOR among the mothers with the GSTP1 AA genotype. However, no statistically significant interactions of GST gene polymorphisms and HNC levels were identified by likelihood ratio testing.

## Discussion

We observed a trend of higher AOR with higher HNC level, suggesting a dose-response relationship between maternal smoke exposure during the first and second trimesters and foetal CHDs. In addition, mothers with deletions in GSTT1and/or GSTM1 tended to have a lower threshold of smoke exposure associated with an elevated risk of CHDs. These findings support our hypothesis that GSTs could modify the association between maternal smoke exposure and CHDs; however, the likelihood ratio analysis we performed did not support a direct interaction between HNCs and GST polymorphisms. This may be because there is no interaction, or perhaps the sample size was too small. Additionally, GST polymorphisms might be more or less likely to interact with HNC, for example, the mothers with an HNC of >0.573 ng/mg and a GSTP1 AG or GG genotype had a much higher risk of CHDs than the mothers with a genotype of GSTP1 AA and the same HNC level.

Accumulating evidence indicates that maternal smoke exposure during pregnancy is potentially associated with an increased risk of CHDs. A recent meta-analysis found a positive association between maternal smoking during pregnancy and CHD risk as a group (RR: 1.11, 95% CI:1.02, 1.21)^3^. In addition, a dose response was observed between the parental smoking category (light, medium, and heavy) and the CHD subtype, e.g., septal defects, conotruncal defects, and left ventricular outflow tract obstructions^7^,^18^,^19^. Our study confirmed that maternal smoke exposure during pregnancy is associated with an increased risk of CHDs. Moreover, the degree of the increased risk of CHDs in our study was much higher than in other studies^3^,^4^,^20^. A very local study population and relatively strict inclusion and exclusion criteria for test cases and controls used in our study, compared to other studies, likely contribute to this difference. Additionally, the method of smoke exposure measurement and the selection of an exposure reference may contribute to the difference. Almost all of the previous epidemiological studies on the correlation between smoke exposure and CHDs used self-reporting to measure smoking exposure. Smoking mothers are likely to under-report or conceal their smoking habits due to social unacceptability or embarrassment and may not accurately recall the amount of smoking even if they have no reservations about reporting it^21^. It is difficult for nonsmoking mothers to accurately measure their exposure to secondhand smoking (SHS), mainly due to the various determinants of SHS, e.g., the duration and frequency of exposure and the concentrations of SHS influenced by the amount of smoking and air exchange^22^,^23^. Although it has been shown that women accurately report their smoking behaviour on birth certificates in some populations, in one study, the Spearman rho correlation coefficient between infant nicotine levels and maternal-reported cigarettes per day was only 0.54 in the third trimester^24^. The Montreal Prematurity Study in Canada also found a low degree of correlation (coefficient: 0.22–0.36) between the maternal HNC and the self-reported number of cigarettes smoked per day in the first and second trimesters^25^. A low accuracy of self-reporting is also illustrated by our finding that a considerable proportion of mothers with a high HNC were present in the unexposed nonsmokers category. If the ‘nonsmokers’ identified through self-reporting are used as the reference group, this group likely contains many participants who are actually exposed to ETS or SHS. Therefore, the risk of CHDs associated with smoking would be underestimated.

Cigarette smoke contains more than 4,000 harmful chemicals, of which approximately 70 are considered “carcinogens”. There is a large amount of evidence suggesting that maternal smoke exposure may seriously affect normal foetal development and increase the risks of low birth weight, premature labour, and congenital anomalies^4^,^26^. Although the mechanisms by which smoke causes CHDs have not yet been clearly elucidated, there are some clues to help us interpret these untoward effects. First, the main smoke by-products, nicotine and carbon monoxide, cross the placental barrier and induce vasoconstriction, resulting in foetal hypoxia^20^. Several animal models have indicated that chronic foetal hypoxia most likely leads to myocardial and ventricle dilation, cardiomyocyte hypertrophy, myocardial hypoplasia, and impaired foetal heart maturation due to increases in the percentage and size of binucleated cardiomyocytes that cannot be differentiated^27^. Second, nicotine can inhibit the expression of the cardiac differentiation marker genes α-actin, desmin, and cTn1, indicating that nicotine can depress early foetal heart development. Third, tobacco smoke not only causes DNA damage to both spermatozoa and oocytes but also causes epigenetic changes. These include alterations in the normal levels of DNA methylation, which can cause subtle changes in gene expression, which is a possible predisposition to congenital anomalies. However, the mechanisms of how male or female germ-cell mutations and epigenetic changes cause CHDs have not yet been clarified^20^.

The variation in the risk of CHDs associated with exposure to toxic agents among different populations suggests that different individuals may have different susceptibilities to the effects of cigarette smoke. In particular, polymorphisms in the GST genes have been extensively studied for smoking-associated diseases. A significant combined effect of maternal smoking and deletions in GSTM1 or GSTT1 in the infant or mother was observed on cleft lip/palate (CL/P) or oral cleft development in case-control studies in the US and the Netherlands^14^,^28^,^29^; however, no gene-smoking interaction effects have been identified in other studies^30^,^31^. A recent case-parent study showed that children with deletions in GSTM1 and GST1 whose parents were exposed to toxins had a higher risk of CHDs than did children with normal copies of GSTM1 and GST1^17^. A significant interaction or joint effect between smoke exposure and the GSTP1 genotype was reported on CL/P^32^; however, conflicting results were observed in another study^33^. Our study suggests that the polymorphisms in the GST genes might influence the association between maternal smoke exposure and CHDs. GSTM1, which is predominantly expressed in the liver, is the primary gene that detoxifies polycyclic aromatic hydrocarbons, the main compounds in cigarette smoke. GSTT1 may play a more global role than GSTM1, as the former detoxifies many types of exogenous compounds and is expressed more broadly, i.e. in erythrocytes, lungs, kidney, brain, skeletal muscle, heart, and small intestine^34^. Mothers with deletions in both GSTM1 and GSTT1 will have a complete loss of both enzymes. In this case, the capacity for xenobiotic detoxification in the body is weakened, and more toxic agents will likely cross the placental barrier, impairing foetal heart development. The findings of our study appear to confirm this point because the mothers with deletions in GSTM1 or GSTT1 tended to have a lower risk threshold of smoke exposure compared to the mothers with normal copies of GSTM1 and GSTT1. However, this finding needs to be confirmed by studies with larger group sizes. For GSTP1, the I105V polymorphism leads to decreased enzyme activity. GSTP1 appears to be important in the detoxification of compounds in cigarette smoke because the gene is widely expressed in the lungs and placenta in the mother and increases in expression in the embryo at 8 and 12 gestational weeks.

Our analysis of the association between maternal smoke exposure, identified by the biomarker HNC, and the risk of foetal CHDs may overcome the bias introduced by the subjectivity of self-reporting in previous studies. We have assessed the potential effects of the GST polymorphisms on the association between maternal smoke exposure and CHDs. Our findings provide important clues to follow in the investigation of the mechanisms of CHD development. However, there are also some limitations in our study. First, the groupings based on HNCs were empirical because there were insufficient objective criteria for establishing reference values; mainly due to a deficiency in the distribution of HNC in the unexposed nonsmoker, as well as active and passive smoking in the Chinese population. Second, the modest group sizes used in our study may have been responsible for the unstable results, such as the wide 95% CI for AOR, and limited the power required to detect the association between maternal smoke exposure and CHDs among the mothers with different GST genotypes. Third, the specific inclusion and exclusion criteria and a very local population in our study may limit the extrapolation of our findings and make direct comparisons to other studies difficult.

## Conclusion

In conclusion, our study suggests that cigarette smoke exposure during pregnancy is associated with an increased risk of foetal CHDs. This association may be modified by polymorphisms in the GST genes. These findings are important for the development of public health strategies and interventions to encourage women of childbearing age, pregnant women, and their families, friends, and colleagues to quit smoking.

## Methods

### Study population and sampling

This GEIOC study used a case-control design. Pregnant women with a gestational age of 14–28 weeks were recruited from six tertiary maternal and child hospitals with the qualification of prenatal screening and diagnosis in China. Foetuses were screened using a systematic ultrasonic examination and targeted echocardiography. Mothers whose foetuses were diagnosed with CHDs but lacked any extra-cardiac abnormalities and mothers whose foetuses were diagnosed without any anomalies were initially chosen as the cases and controls, respectively. Each live birth was examined within seven days to confirm the prenatal diagnosis. Autopsy was the preferred method to confirm the prenatal diagnoses for cases of terminated foetus. If an autopsy was not possible, the static and dynamic echocardiography images were reviewed by ultrasound specialists for the final diagnosis. Additionally, all live births were followed up for three months to confirm their final diagnoses. The details regarding the recruitment process have been well-described in two published reports^7^,^35^,^36^. Multiple exclusion criteria were used for the study: (1) a woman with multiple foetation; (2) a mother with a child who was selected as a control but exhibited congenital abnormalities after birth; (3) a mother with a child with heart abnormalities but an unclear diagnosis; and (4) CHD cases associated with a known syndrome or genetic abnormality. Additionally, isolated cardiomyopathies, a single umbilical artery, and rhythm disorders (i.e., atrioventricular blocks and Wolff-Parkinson-White syndrome) were not considered to be CHDs.

**Figure 2:**
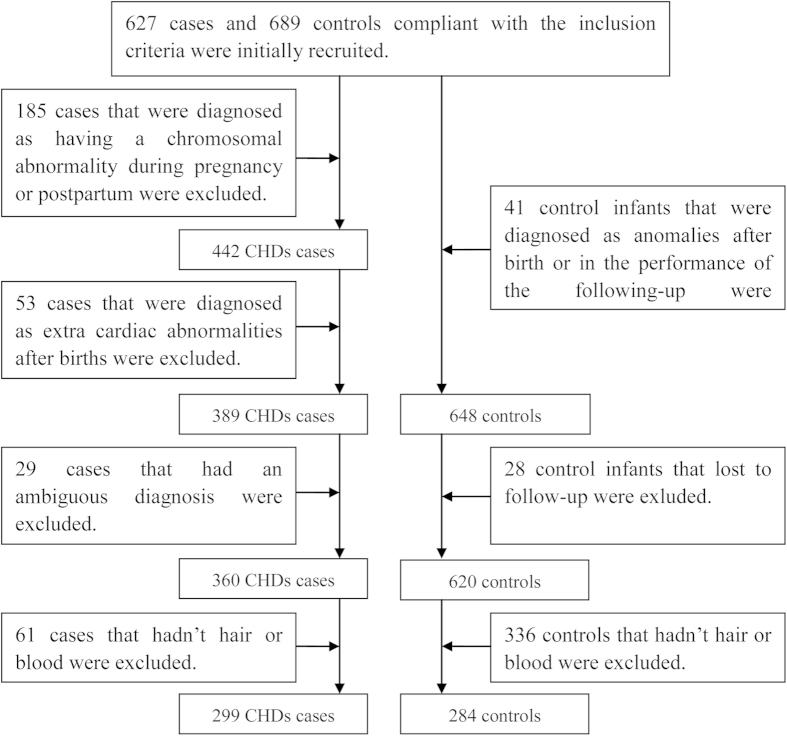
Flow chart of inclusion and exclusion of the case and control subjects.

During the study period from Feb. 2010 to Oct. 2012, 627 cases and 689 controls that were compliant with the inclusion criteria were initially recruited into the GEIOC program. During recruitment, each pregnant mother was interviewed using a structured questionnaire. Several types of biological samples (e.g., maternal hair, maternal blood, urine, amniotic fluid, and placenta) were collected during pregnancy or postpartum after obtaining the mother’s consent. Thus, an epidemiological and biological databank for CHD cases and controls was established. In the present study, the participants were chosen from this databank. Due to the exclusion criteria, 267 cases and 69 controls were excluded from the study. Additionally, 61 cases and 336 controls were excluded due to a lack of maternal hair or blood samples. Therefore, 299 cases and 284 controls were included in the study. The flowchart of case and control inclusion and exclusion is shown in Fig. 2.

The study was approved by the Medical Ethics Committee of Sichuan University (No. 2010004). Written informed consent was obtained from all subjects. All experiments involving human subjects and tissues were performed in accordance with guidelines approved by Sichuan University.

### Case classification

The CHD cases were classified into six subtypes based on the anatomic lesion as follows: (1) septal, including atrial septal defects, ventricular septal defects, and endocardial cushion defects; (2) conotruncal, including transposition of the great arteries, tetralogy of Fallot, truncus arteriosus, and double outlet right ventricle; (3) right-sided obstructive, including pulmonary valve stenosis, pulmonary atresia, tricuspid atresia, and Ebstein anomalies; (4) left-sided obstructive, including aortic valve stenosis, hypoplastic left heart syndrome and variants, coarctation of the aorta, and interrupted aortic arch; (5) anomalous venous return, including total and partial anomalous pulmonary or systematic venous return; and (6) others, including single ventricle, heterotaxia, and other cardiac structural abnormalities.

### Exposure Measurements

Self-reported measurements: Information on maternal smoke exposure was collected through specific questions, including whether the pregnant woman or her husband smoked and their average daily smoking amount, whether other smokers were physically near the pregnant woman and their average daily smoking amount, and whether the mother actively avoided others or environments that exposed her to smoke. The subjects were classified based on the answers into five groups: (1) unexposed nonsmokers, which referred to nonsmoking mothers who reported that they had no exposure to smoking environments; (2) slight passively exposed nonsmokers, which referred to nonsmoking mothers who reported that they were exposed to smoking environments but actively avoided these areas; (3) moderate passively exposed nonsmokers, which referred to nonsmoking mothers who reported that they were exposed to smoking environments with <10 cigarettes per day and did not actively avoid these environments; (4) severe passively exposed nonsmokers, which referred to non-smoking mothers who reported that they were exposed to smoking environments with ≥10 cigarettes per day and did not actively avoid these environments; and (5) active smokers.

Biomarker measurements: Maternal hair samples were collected soon after the questionnaire was collected. Pencil-width locks of hair were cut from the posterior vertex of the scalp using a fine pair of scissors, placed in individually labelled sterile envelopes and stored at −20 °C until analysis. Because the average hair growth rate is approximately 1 cm per month, approximately 3- to 4-cm-long segments were cut close to the scalp in the first and second trimesters. Before analysis, the hair samples were washed with ethanol, dichloromethane, and distilled water in turn to remove any environmental contamination and then dried overnight. The hair analysis was conducted in a single-blinded manner in which the testers did not know the identities of the samples. The hair nicotine analysis was performed using a Gas Chromatograph-Mass Spectrometer (GC-MS). After being cut and minced into 1- to 2-mm-long pieces, 0.01–0.05 g of hair sample was accurately weighed on an analytical balance, placed in a test tube, mixed with 2 ml of a 1.0 mol/L NaOH solution and incubated at 37 °C overnight (12 hours). The digestion solution was extracted with 2.0 ml of dichloromethane by vortex mixing for 1 min. The extract was centrifuged, and 1.5 ml of the organic phase was transferred into a polypropylene tube containing 500 μl of methanol/25 mM HCl. The sample was evaporated to dryness under a stream of nitrogen at 40 °C. The residue was reconstituted in 200 μl of methylene chloride containing 2 μg/ml of quinoline (the internal standard), and 1 μl of the solution was injected into the GC-MS. The HNC results were expressed in nanograms of nicotine per milligram of hair, with a detection limit of 0.01 ng/mg. Further details regarding the hair nicotine analysis are described elsewhere^37^.

### Genotyping

Genomic DNA was extracted from peripheral blood leukocytes using the QIAamp DNA Blood Mini Kit (Qiagen, Germany) according to the manufacturer’s instructions. The GSTT1 and GSTM1 genotypes were determined using GSTT1-specific primers (forward: 5′ TTCCTTACTGGTCCTCACATCTC 3′; reverse: 5′ TCACCGGATCATGGCCAGCA 3′) or GSTM1-specific primers (forward: 5′ GAACTCCCTGAAAAGCTAAAGC 3′; reverse: 5′ CTTGGGCTCAAATATACGGTGG 3′) using a multiplex PCR approach. As an internal control, the β-globin gene was amplified in the same reaction (forward: 5′ CAACTTCAT CCACGTTCACC 3′; reverse: 5′ GAAGAGCCAAGGACAGGTAC 3′). The PCR reaction was performed in a final volume of 25 μl containing 1 μl (20 ng) of purified genomic DNA, 1 μl of forward primer, 1 μl of reverse primer (5 pmol of each), 2 μl of 2.5 mM dNTPs, 2.5 μl of 10 × buffer, 2 μl of 25 mM MgCl_2_, and 0.2 μl of HSTaq DNA Polymerase (TaKaRa Corp., Dalian, China). The PCR procedure used the following conditions: 95 °C for 3 min, followed by 30–36 cycles of 95 °C for 30 s, 64 °C for 45 s, and 72 °C for 1 min, with a final extension at 72 °C for 5 min. The amplification was performed with a thermal Cycler C1000 (Bio-Rad, CA, USA). The β-globin internal standard product was 268 bp. The GSTM1 and GSTT1 products were 215 bp and 480 bp, respectively. A 292 bp fragment containing the GSTP1 polymorphism was amplified (forward: 5′ATCCTTCCACGCACATCCTCT3′; reverse: 5′ AAGCCCCTTTCTTTGTTCAGC3′) in the same procedure except that an annealing temperature of 59 °C was used. The product was then digested with *HpyCH4IV* (NEB, USA) to detect the three genotypes: AA (292 bp fragment), AG (292, 215, and 77 bp fragments) and GG (215 and 77 bp fragments). All PCR products and digestion fragments were electrophoresed on a 3% agarose gel containing GELVIEW, and the results were visualized on the Bio-Rad Gel-Doc 1000 apparatus (Bio-RAD, CA, USA).

### Data analysis

A case-control analysis was performed to assess the associations between maternal smoke exposure and CHDs as well as the effect of GST gene polymorphisms on the associations. The potential confounders were those correlated with both the main determinant and CHDs. These confounders included maternal age (<25, 25–34, or ≥35years), maternal education(<12, 12–15, or ≥16 years), gestational age (14–22 or 23–28 weeks), and maternal drinking (often (≥1 time(s)/week), occasional(<1 time/week), or never) during the three months prior to the first trimester, and maternal ppBMI (<18.5, 18.5–24.0, or ≥24.0). Based on the percentiles of the HNCs (P20, P40, P60, and P80) for the combined sample, the smoke exposure was divided into 5 levels: ≤0.117, 0.117–0.213, 0.213–0.319, 0.319–0.573 and >0.573 ng/mg.

T-test was used to test the difference in the logarithm of HNC between the cases and controls. Differences in the proportions between the cases and controls regarding potential factors and GST genotypes were tested using the Chi-square test. Multivariable dichotomous logistic models were used to assess the adjusted associations between CHDs and HNC categories. The models were expressed as: Logit(P) = HNC + {adjusters} + ε. In the models, the presence or absence of any CHD or its subtype was the dependent variable, and the HNC level was the main independent variable. HNC was set as a categorical variable, and the ≤0.117 ng/mg level was set as the reference group. The potential confounders were chosen as adjusters and added as categorical variables into the model. The results of these models are shown in Table 3. In addition, the adjusted associations between any CHDs and HNC level among GSTM1, GSTT1, and GSTP1 polymorphisms were evaluated by logistic models. These models were expressed as: Logit(P) = HNC + GST + GST*HNC + {adjusters} + ε. The significance of the GST*HNC terms was tested by hierarchical likelihood ratio testing. The results of these models are shown in Table 4.

All of the statistical analyses were performed using SAS 9.0 software (SAS Institute, Cary, NC, USA). Two-tailed values of P<0.05 and 95% CIs excluding 1.00 were considered to be statistically significant.

## Additional Information

**How to cite this article**: Li, X. *et al.* Modification of the association between maternal smoke exposure and congenital heart defects by polymorphisms in glutathione S-transferase genes. *Sci. Rep.* **5**, 14915; doi: 10.1038/srep14915 (2015).

## Figures and Tables

**Table 1:** Maternal characteristics of the case and control groups.

	Case group No.(Pct)	Control group No.(Pct)	χ^2^(P)
Maternal age(yrs)			5.391(0.0680)
<25	63(21.07)	41(14.44)	
25–34	204(68.23)	202(71.12)	
≥35	32(10.70)	41(14.44)	
Maternal education (yrs)			44.007 (<.0001)
<12	112(37.46)	42(14.79)	
12–15	135(45.15)	148(52.11)	
≥16	52(17.39)	94(33.10)	
Gestational weeks^a^			7.72(0.0505)
14–22	114(38.13)	131(46.13)	
23–28	185(61.87)	153(53.87)	
Maternal drinking^b^			
Often	7(2.34)	8(2.82)	14.40(0.0007)
Occasional	47(15.72)	81(28.52)	
Never	245(81.94)	195(68.66)	
ppBMI^c^			6.82(0.0330)
<18.5	85(28.43)	63(22.18)	
18.5–24.0	184(61.54)	203(71.48)	
≥24.0	30(10.03)	18(6.34)	
GSTT1			
Normal	153(51.17)	160(56.34)	1.56 (0.2110)
Deleted	146(48.83)	124(43.66)	
GSTM1			
Normal	139(46.49)	140(49.30)	0.46(0.4976)
Deleted	160(53.51)	144(50.70)	
GSTT1 and GSTM1			
One or both normal	221(73.91)	208(73.24)	0.034(0.8537)
Both deleted	78(26.09)	76(26.76)	
GSTP1 (rs1695)			
AA	202(67.56)	209(73.59)	2.55(0.1104)
AG or GG	97(32.44)	75(26.41)	

^a^Gestational weeks were determined on the basis of gestational age in weeks at recruitment.

^b^Maternal drinking: the frequency of maternal drinking during the three months prior to the first trimester is classified into three groups, often: ≥1 time(s)/week; occasional: <1 time/week); or never.

^c^ppBMI: pre-pregnancy body mass index.

**Table 2:** Descriptive statistics of HNC in the case and control groups.

HNC^a^(ng/mg)	Case group	Control group
N	299	284
Geometric Mean	0.369	0.171
Mean ± Standard Deviation	0.606 ± 0.805	0.269 ± 0.426
Stratified by reported smoking level:		
Unexposed non-smokers	0.504 ± 0.623	0.245 ± 0.225
Slight passively exposed non-smokers	0.647 ± 0.854	0.298 ± 0.618
Moderate passively exposed non-smokers	0.583 ± 0.559	0.319 ± 0.218
Severe passively exposed non-smokers	0.927 ± 1.423	0.289 ± 0.208
Active smokers	0.715 ± 0.499	0.142 ± 0.118
Percentiles		
P0	0.021	0.012
P20	0.174	0.085
P25	0.213	0.096
P40	0.298	0.149
P50	0.368	0.180
P60	0.435	0.229
P75	0.648	0.302
P80	0.784	0.346
P100	7.230	5.285

^a^HNC: Hair Nicotine Concentration.

**Table 3:** Association between maternal HNC levels and CHDs.

HNC^a^(ng/mg)	Case	Control	COR^b^(95%CI^d^)	AOR^c^(95%CI^d^)
Any CHDs				
≦0.117	36	81	Ref.	Ref.
0.117–0.213	38	78	1.10(0.63,1.90)	0.91(0.51,1.65)
0.213–0.319	56	60	2.10(1.23,3.59)	1.94(1.09,3.46)
0.319–0.573	78	40	4.39(2.54,7.58)	4.37(2.42,7.88)
>0.573	91	25	8.19(4.53,14.8)	6.83(3.64,12.80)
Septal defect				
≦0.117	10	81	Ref.	Ref.
0.117–0.213	16	78	1.66(0.71,3.88)	1.34(0.54,3.30)
0.213–0.319	22	60	2.97(1.31,6.73)	2.94(1.22,7.09)
0.319–0.573	26	40	5.26(2.31,11.97)	6.24(2.52,15.44)
>0.573	26	25	8.42(3.58,19.83)	7.84(3.13,19.68)
Conotruncal defect				
≦0.117	16	81	Ref.	Ref.
0.117–0.213	15	78	0.97(0.45,2.10)	0.89(0.40,1.97)
0.213–0.319	24	60	2.03(0.99,4.14)	1.91(0.91,4.03)
0.319–0.573	37	40	4.68(2.33,9.41)	5.05(2.41,10.59)
>0.573	46	25	9.32(4.51,19.22)	7.76(3.61,16.66)
Left-sided obstructive				
≦0.117	8	81	Ref.	Ref.
0.117–0.213	6	78	0.78(0.26,2.35)	0.68(0.21,2.19)
0.213–0.319	10	60	1.69(0.63,4.53)	1.53(0.52,4.51)
0.319–0.573	14	40	3.54(1.37,9.14)	4.89(1.69,14.15)
>0.573	17	25	6.89(2.66,17.85)	5.81(2.05,16.48)
Right-sided obstructive				
≦0.117	6	81	Ref.	Ref.
0.117–0.213	11	78	1.90(0.67,5.40)	1.59(0.55,4.63)
0.213–0.319	10	60	2.25(0.78,6.53)	2.12(0.71,6.31)
0.319–0.573	15	40	5.06(1.83,14.04)	5.13(1.78,14.80)
>0.573	21	25	11.34(4.12,31.20)	9.87(3.45,28.23)
Anomalous pulmonary venous return				
≦0.117	2	81	Ref.	Ref.
0.117–0.213	4	78	2.08(0.37,11.66)	1.61(0.27,9.79)
0.213–0.319	8	60	5.40(1.11,26.34)	5.08(0.93,27.85)
0.319–0.573	9	40	9.11(1.88,44.16)	12.66(2.25,71.19)
>0.573	11	25	17.82(3.70,85.81)	12.34(2.31,65.88)

^a^HNC: Hair Nicotine Concentration.

^b^COR: Crude Odds Ratio.

^c^AOR: Adjusted Odds Ratio, adjusted for maternal age, maternal education, gestational weeks, drinking, and ppBMI.

^d^CI: confidence interval.

**Table 4:** Associations between HNC level and CHDs among mothers with different GST genotypes.

	HNC^a^ (AOR^b^(95% CI^c^))					Interaction Test^d^
	<0.117 ng/mg	0.117–0.213 ng/mg	0.213–0.319 ng/mg	0.319–0.573 ng/mg	>0.573 ng/mg	G^2^(P-value)
GSTM1						1.556(0.817)
Normal	Ref.	1.05(0.45,2.46)	1.60(0.69,3.69)	4.46(1.90,10.46)	6.01(2.37,15.26)	
Deleted	1.14(0.49,2.65)	0.91(0.39,2.15)	2.76(1.18,6.45)	4.97(2.11,11.73)	8.51(3.50,20.70)	
GSTT1						3.050(0.550)
Normal	Ref.	0.80(0.36,1.78)	1.48(0.66,3.32)	3.51(1.56,7.88)	8.28(3.44,19.97)	
Deleted	1.02(0.44,2.36)	1.13(0.48,2.67)	2.61(1.16,5.86)	5.63(2.42,13.14)	5.60(2.31,13.55)	
GSTM1 and GST1						2.659(0.616)
One or both normal	Ref.	0.95(0.48,1.88)	1.60(0.81,3.15)	4.23(2.13,8.41)	7.44(3.51,15.78)	
Both deleted	0.89(0.34,2.29)	0.70(0.26,1.90)	2.89(1.17,7.14)	4.20(1.60,11.06)	5.13(1.97,13.37)	
GSTP1						6.967(0.138)
AA	Ref.	0.93(0.46,1.89)	2.08(1.03,4.20)	4.39(2.19,8.81)	4.97(2.42,10.21)	
AG or GG	1.32(0.53,3.26)	1.16(0.46,2.89)	2.16(0.92,5.06)	6.04(2.24,16.25)	38.53(8.17,181.84)	

^a^HNC:Hair Nicotine Concentration.

^b^AOR: Adjusted Odds Ratio, adjusted for maternal age, maternal education, gestational weeks, drinking, and ppBMI.

^c^CI :Confidence Interval.

^d^Interaction terms of GST*HNC were tested by hierarchical likelihood ratio testing, and the statistic G^2^ follows a chi-square distribution with 4 degrees of freedom.
